# Process of cerebral edema in the infarct core after reperfusion: A case report

**DOI:** 10.1097/MD.0000000000029810

**Published:** 2022-06-30

**Authors:** Liebiao Peng, Rongfei Wang

**Affiliations:** a Department of Neurology, The Affiliated Brain Hospital of Guangzhou Medical University, Guangzhou, China; b The Second School of Clinical Medicine, Guangzhou University of Chinese Medicine, Guangzhou, China.

**Keywords:** cerebral edema, cerebral infarction, hypoattenuation, reperfusion

## Abstract

It is generally believed that cerebral infarction shows hypoattenuation on computed tomography (CT) scans 12 to 24 hours after onset. Cerebral edema affects the occurrence of hypoattenuation, originating from the inner edge of the infarct and extending to the core. When reperfusion occurs in the infarct, the process of cerebral edema changes significantly, affecting the imaging of cerebral infarction on CT scans. This article focused on the mechanism of cerebral edema to provide a new perspective for understanding the impact of reperfusion on cerebral infarction.

**Patient concerns:**

We describe the case of a 77-year-old man who presented with an acute onset of right limb weakness with speech difficulties 10 hours before the visit. He had been diagnosed with atrial fibrillation 4 months ago. During the acute phase of infarction, the central area of the hypoattenuated infarct appears as isodensity on CT scans in this case.

**Diagnoses:**

The patient was diagnosed with acute cerebral infarction, cardiogenic cerebral embolism, and spontaneous recanalization of left middle cerebral artery occlusion.

**Interventions:**

Metoprolol was given to control the ventricular rate. The patient received blood pressure control, symptomatic management, and rehabilitation treatments.

**Outcomes:**

Finally, the patient became alert.

**Lessons:**

Cerebral edema originating directly in the infarct core after reperfusion could lead to a significantly accelerated edema process and imaging evolution, causing more severe cerebral damage. In such a case, the patient should not receive antiplatelet and anticoagulant therapy in order to prevent bleeding conversion.

## 1. Introduction

Cerebral infarction is the most common cerebrovascular disease, accounting for about 70% of all cerebrovascular diseases. Symptoms of cerebral infarction include cerebral tissue ischemia, hypoxia, and necrosis caused by cerebrovascular obstruction. Ischemia and hypoxia further lead to dysfunction of the Na^+^–K^+^ exchange pump on the cell membrane, followed by increased membrane permeability and cerebral edema.^[1]^ Cerebral edema indicates the entry of new substances called edema fluids. The edema fluids are not oozed from the nonperfused infarct core but from the peripheral perfused area adjacent to the infarct.^[2]^ Therefore, the edema appears first at the inner edge of the infarct.^[3,4]^ It takes time for the edema fluids to travel from the edge of the infarct to the core. This infiltration course usually causes a peak of edema after 3 to 5 days. Cerebral edema also affects the imaging of brain tissue. In addition, cerebral infarction usually does not show hypoattenuation on computed tomography (CT) scans until 12 to 24 hours of onset.^[5]^

The process of cerebral edema will change significantly when the occluded blood vessels are recanalized after ischemic injury. A case has been reported in which the cerebral infarct with spontaneous reperfusion showed typical hypodensity on CT scans at the fourth hour of onset and restored to isodensity at the 5.5th hour.^[6]^ According to the authors, because of the increased permeability of the blood–brain barrier (BBB) caused by ischemic injury and the propensity of reperfusion to drive the flow of blood to the infarct core, the edema fluids could reach the extravascular space directly in the infarct core from the capillaries, thereby leading to cerebral edema. This process was faster than the infiltration course of edema fluids traveling from the peripheral perfused area adjacent to the nonperfused infarct. Therefore, in the reperfusion state, the process of cerebral edema is significantly accelerated, and the evolution of cerebral ischemia is also greatly promoted on CT imaging.

However, there is no clinical evidence of cerebral edema caused by reperfusion originating in the infarct core. We report a case of cerebral infarction with spontaneous reperfusion. During the acute phase of infarction, the central area of the hypoattenuated infarct appears as isodensity on CT scans in this case. This characteristic suggests that cerebral edema originates in the infarct core after reperfusion.

## 2. Case report

A 77-year-old man presented to our institute with an acute onset of right limb weakness with speech difficulties 10 hours before the visit. The patient had been diagnosed with atrial fibrillation 4 months ago, did not take medication regularly, and had no history of hypertension, diabetes, hematopathy, smoking, or alcohol abuse. A neurological examination was performed at the time of admission. Subsequently, the patient presented somnolence with bilateral pupils sensitive to the light and bilateral eyeballs gazing to the left. The eyeballs could return to the midline. He had facial droop on the left side and pronounced only monosyllables with symptoms of tingling and numbness on the right side. The muscle strength of the right upper limb was grade 1 and that of the right lower limb was grade 2, and the Babinski sign was positive on the right side. The National Institutes of Health Stroke Scale (NIHSS) score was 18.

**Figure 1.**
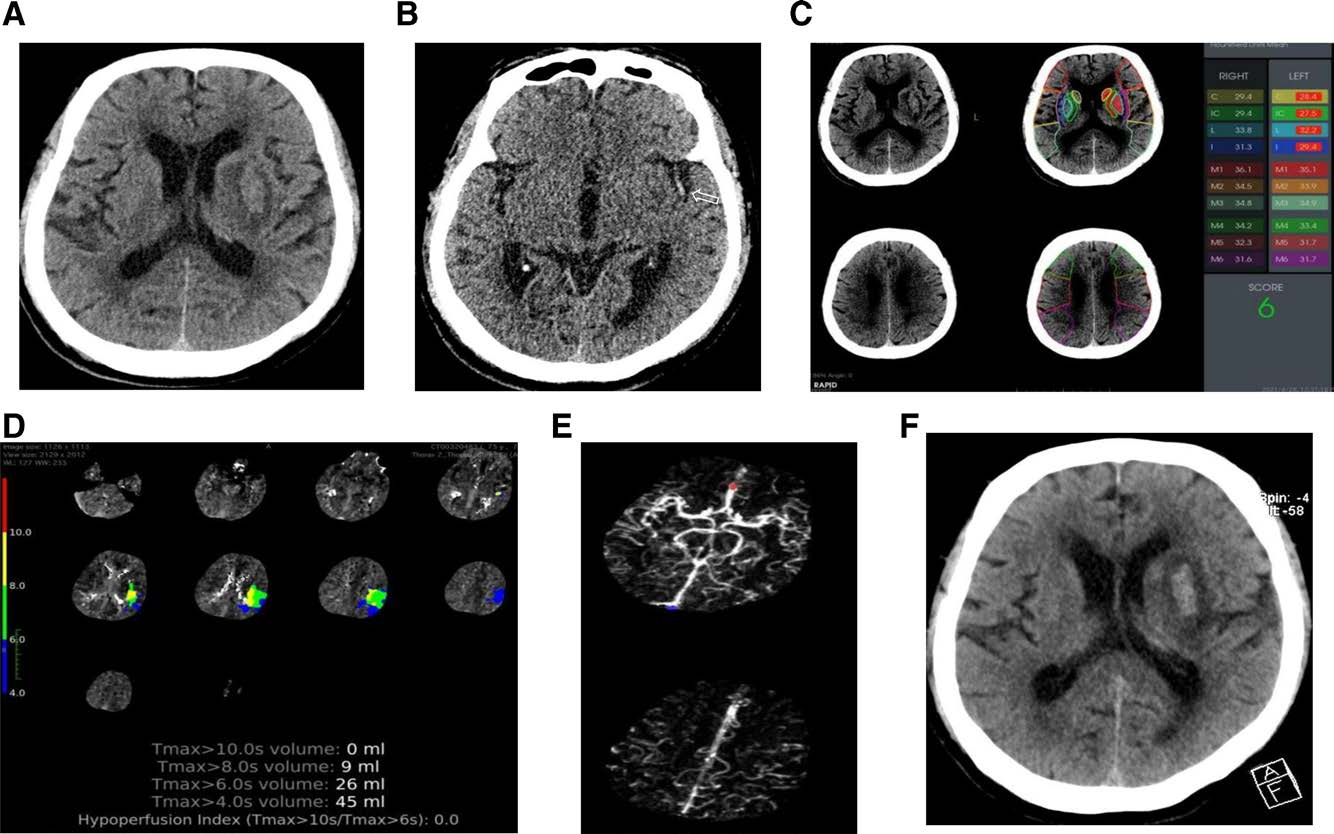
CT images of a patient with infarct in the left cerebral hemisphere. (A) Hypoattenuation in the left basal ganglia and isodensity in the central area (lenticular nucleus) of the hypoattenuation region at the 10th hour of onset. (B) Hyperdensity sign (white arrow) at the MCA M2 segment. (C) RAPID CT scans showed the infarct in the lenticular nucleus, caudate nucleus, internal capsule, and insula, and the RAPID score was 6. (D) Hypoperfusion in the territory of the upper trunk of the left MCA on perfusion CT scans. (E) CT angiography demonstrated the left MCA M1 segment and slightly fewer distal branches than the right. (F) After 3 days of onset, the hypoattenuation region’s center area (lenticular nucleus) changed from isodensity to slightly higher density on CT scans. CT = computed tomography, MCA = middle cerebral artery.

According to the history, symptoms, signs, and examination images, the patient was diagnosed with acute cerebral infarction, cardiogenic cerebral embolism, and spontaneous recanalization of left middle cerebral artery occlusion. Antiplatelet and anticoagulant drugs were not administered, but metoprolol was given to control the ventricular rate. The patient also received blood pressure control, symptomatic management, and rehabilitation treatments. On the third day of onset, the patient maintained obtundation and slurred speech. The muscle strength of the right limb increased to grade 3, and the NIHSS score dropped to 12. The second head CT scans showed mild hyperattenuation in the central area of the infarct. On the ninth day of onset, the patient became alert. The muscle strength of the right limb increased to grade 4, whereas the NIHSS score decreased to 6 (Fig. 1A–F). Radiofrequency ablation was planned to deal with his atrial fibrillation.

## 3. Discussion and conclusions

The CT value of brain tissue correlates with the contents of protein and lipid. Rieth et al^[5]^ produced vasogenic edema by frostbiting rhesus monkeys’ brain tissue and observed the relationship between brain tissue edema and CT value. The results showed that CT value decreased by 10% at 6 hours after injury, by 35% on day 5, and then gradually increased, returning to the baseline level by day 20. Further histological analysis revealed a 30% reduction in protein and fat contents and an 11.3% increase in water content on the injured side when compared to the healthy side. Cerebral edema indicated the entry of new substances (edema fluids) that were not oozed out from the nonperfused infarct core but rather from the peripheral perfused area adjacent to the infarct.^[2]^ Pathological studies in animal experiments and clinical imaging studies confirmed the edema at the inner edge of the infarct before the infarct core, thereby supporting the viewpoint conveyed earlier.^[3,4]^

Water and ions travel from the peripheral perfused area to the nonperfused infarct core in case of ischemic brain tissue injury. This infiltration course takes time to complete, resulting in a peak of cerebral edema after 3 to 5 days. Thus, CT findings typically show hypoattenuation 12 hours after onset. After reperfusion of the ischemic brain tissue, the reperfused blood directly reaches the infarct core, and the water, ions, proteins, and lipids in the blood directly arrive at the extravascular space through the damaged BBB, without using the above-mentioned infiltration course. Therefore, reperfusion significantly promotes the process of cerebral edema and hypoattentuation in the infarct region.

Ischemic injury leads to BBB damage, increasing capillary permeability. The degree of BBB damage determines the molecular weight of the substances that can penetrate BBB. The more severe the damage, the higher the molecular weight of the substances penetrating BBB. The molecular weight of water and various ions is <100 Dalton (Da). Nonionic iodine contrast agents commonly used clinically, such as Iodol, have molecular weights between 600 and 1500 Da.^[7]^ Moreover, the molecular weights of albumin and hemoglobin are about 6900 and 65,000 Da, respectively. When the BBB is mildly damaged, only low-molecular-weight substances, such as water and various ions, can permeate into the extravascular space in large quantities, thereby resulting in ionic edema. As water content increases, the CT value of brain tissue decreases, showing hypoattentuation. When the BBB damage degree is moderately damaged, the iodine contrast agent can penetrate out of the vessel. As the damage worsens, large molecules such as albumin can also pass through the damaged BBB. When the BBB is severely damaged and the tight connections between cells are broken, hemoglobin can also leak out from the blood vessel, that is, blood extravasation.^[8]^ When the amount of iodine, albumin, or hemoglobin in the brain tissue increases, the CT value of the brain tissue increases correspondingly.

As the neuron-rich gray matter is more sensitive to ischemia and hypoxia than nerve fiber-rich white matter, the BBB of gray matter is more severely impaired than that of white matter under the same ischemic injury. The more severe the ischemic injury, the higher the molecular weight of the substances that can penetrate the BBB. When brain tissue with the same degree of ischemic injury is reperfused, the white matter only allows low-molecular-weight substances such as water and ions to penetrate the impaired BBB, causing hypoattenuation on CT scans. On the other hand, the gray matter allows high-molecular-weight proteins and lipids to permeate the BBB, thereby causing the CT value to return to isodensity. As in the case reported here, the lenticular nucleus is isodense, whereas its surrounding areas, internal capsule, and outer capsule are hypodense on CT scans. Our case supports the idea that cerebral edema occurs directly within the infarct core after reperfusion. When albumin and other macromolecules travel from the peripheral perfused area to the infarct core, the protein content first increases at the inner edge of the infarct, leading to isodense CT value.

In cases of cerebral infarction with mechanical recanalization, the rebound of postoperative CT value is very common, accounting for >86%, and indicates blood extravasation and/or iodine agent staining.^[9]^ The iodine agent staining the infarct is generally absorbed within 24 hours.^[10]^ The iodine agent is rapidly distributed throughout the body in circulation within ~10 minutes after injection into a blood vessel. In normal kidney function, the clearance half-life of iodine agent is about 2 hours, and about 90% of the iodine agent is excreted through the kidneys within 24 hours.^[7]^ As a result of renal excretion, the concentration of iodine agent in the plasma decreases in relation to the concentration in the tissue space, so the iodine agent refluxes into the blood vessel along the concentration gradient, which comprises the process of iodine agent absorption. In the case we reported, without the injection of the iodine agent, the CT value also increased after reperfusion. We considered the proteins and lipids extravasated from a blood vessel and increased their concentration in the infarct, which led to an increase in the CT value. As the molecular weight of the iodine agent is lower than that of the proteins and lipids, we suggest that there would have been iodine agent staining in the infarct if the patient had been injected with the iodine agent. This patient experienced more severe cerebral ischemic damage than patients with iodine agent staining alone, and precautions were required to prevent bleeding conversion. Therefore, the patient did not undergo antiplatelet and anticoagulant therapy.

In conclusion, cerebral ischemia injury leads to the destruction of BBB and then cerebral edema beginning at the inner edge of the infarct. After reperfusion, cerebral edema occurs directly in the infarct core, and its process is significantly accelerated.

## Author contributions

Conceptualization: Liebiao Peng, Rongfei Wang.

Supervision: Rongfei Wang.

Writing – original draft: Liebiao Peng, Rongfei Wang.
